# Dengue hemorrhagic fever and severe thrombocytopenia in a patient on mandatory anticoagulation; balancing two life threatening conditions; a case report

**DOI:** 10.1186/1471-2334-12-272

**Published:** 2012-10-26

**Authors:** Champika Gamakaranage, Chaturaka Rodrigo, Sincy Samarawickrama, Dilushi Wijayaratne, Malaka Jayawardane, Panduka Karunanayake, Saroj Jayasinghe

**Affiliations:** 1 University Medical Unit, National Hospital of Sri Lanka, Colombo, Sri Lanka; 2 Department of Clinical Medicine, Faculty of Medicine, University of Colombo, Colombo, Sri Lanka

## Abstract

**Background:**

Managing a severe dengue infection is a challenge specially when complicated by other comorbidities. We report a patient with dengue haemorrhagic fever and spontaneous bleeding who required mandatory anticoagulation for a prosthetic mitral valve replacement. This is the first case report in published literature describing this therapeutic dilemma.

**Case presentation:**

A fifty one year old Sri Lankan woman was diagnosed with dengue haemorrhagic fever with bleeding manifestations. During the critical phase of her illness, the platelet count dropped to 5,000/ɥl. She was also on warfarin 7 mg daily following a prosthetic mitral valve insertion. In managing the patient, the risk of bleeding had to be balanced against the risk of valve thrombosis without anticoagulation. Warfarin was withheld when the platelet count dropped to 100,000/ɥl and restarted when it recovered above 50,000/ɥl. The patient was off anticoagulation for 10 days.

**Conclusions:**

We managed this patient with close observation and continuous risk benefit assessments of management decisions. However, experience with one patient cannot be generalized to others. Therefore, it is essential that clinicians share their experiences in managing such difficult patients.

## Background

Dengue haemorrhagic fever is a potentially lethal illness that is universally prevalent in the tropics [1]. Severe infection is characterized by a ‘leakage phase’ (or critical phase) usually lasting 48 hours following an initial febrile phase [2]. During the leakage phase, an increase in capillary permeability leads to extravasation of fluid and haemoconcentration. During the latter stages of febrile phase and early leakage phase (or even later), there is a steady drop in platelet count. At occasions, it can drop as low as 3000/μl in previously healthy individuals (normal platelet count in a healthy adult: 150,000 – 400,000/ μl). The exact mechanism of this drop is unclear but presumed to be immunological. The low platelet count leaves the patient at a significant risk of spontaneous bleeding. The management is further complicated by pre-existing co-morbidities that interfere with the usual therapeutic guidelines. We report a patient with severe dengue infection with very low platelet counts and haemorrhagic manifestations who was also on mandatory anticoagulation with warfarin for a prior prosthetic mitral valve replacement.

## Case presentation

A fifty one year old Sri Lankan woman, presented with fever for three days and constitutional symptoms such as arthralgia, myalgia and headache. There was no specific focus for an infective process such as a urinary or a respiratory tract infection. She was a diagnosed patient with tight mitral stenosis (following rheumatic carditis), for which a metallic mitral valve replacement had been done eight years ago. She was on lifelong anticoagulant therapy with target INR (International Normalized Ratio) maintained with 7 mg of warfarin per day. Her other medication included; digoxin for atrial fibrillation, penicillin prophylaxis for rheumatic valvular disease and a combination of captopril, spiranolactone and furosemide for heart failure. Physical examination showed pallor, dental caries and ankle oedema without any peripheral stigmata of infective endocarditis. On admission, she was hemodynamically stable with a pulse rate of 82 beats per minute and a blood pressure of 110/60 mmHg. There was no postural drop in blood pressure or a narrowed pulse pressure to indicate intravascular volume depletion. On auscultation, the metallic first heart sound and a loud pulmonary component of the second heart sound were heard. Lungs had a few bilateral basal crackles. Rest of the examination was normal.

The provisional diagnosis was dengue fever as the clinical picture was typical of the infection. However, measures were taken to exclude an alternative infective process such as infective endocarditis. Her admission coincided with a dengue epidemic in the area. Strict monitoring of vital parameters and appropriate fluid management was initiated according to the national guidelines on dengue [2].

The initial full blood counts showed a leucopenia and a trend of dropping platelet counts confirming our suspicion. The dengue antibodies assessed by IgM antibody capture ELISA (MAC- ELISA) were positive indicating an acute infection. On day 3 after admission (date of admission taken as day 0), her fever subsided but there was evidence of a small right sided pleural effusion indicating the onset of plasma leakage and the start of critical/leakage phase. The platelet count that was already low was expected to hit a nadir between days 4–5 (within 48 hours after the start of critical phase). The patient was at high risk of internal haemorrhage. The usual management protocol at this time calls for fluid restriction and observation for bleeding manifestations. Prophylactic platelet transfusions are discouraged. However, this particular patient had the complication of a mitral valve replacement needing mandatory anticoagulation. Stopping anticoagulation had the potential life threatening complication of valve thrombosis while continuing warfarin had the risk of a torrential internal haemorrhage aided by an already low platelet count. In the absence of guidelines or even previous case reports with similar experience, we managed the patient with institutional expert opinion.

Fever and vomiting was treated symptomatically with paracetamol and domperidone. Spiranolactone, captopril and furosemide were temporarily withheld since admission. Once the platelet count dropped below 100 × 10^3^/μl, warfarin was withheld. Digoxin was continued at its usual dose of 0.125 μg three times daily. The rationale for stopping warfarin was to keep the patient off it during the critical phase until the platelet count picked up. However, as warfarin has a long half life, it was necessary to stop it at least two to three days before the anticipated onset of critical phase. The plan was to restart warfarin as soon as the platelet count was back within ‘safe’ margins. It was anticipated that the critical phase would only last for 48 hours (which is the typical clinical picture). It was a race against time and she was closely monitored both clinically and echocardiographically for evidence of valve thrombosis.

Three days after stopping warfarin she entered the critical leakage phase (heralded by the detection of the pleural effusion). During this time she developed gum bleeding, petechiae and haemorrhagic blebs reaching a maximum size of 2 × 2 cm at venepuncture sites in both arms. During the critical phase, the platelet count dropped to 5,000/μl. It was decided to transfuse her with one unit of red cell concentrate and four units of platelet concentrates at this time. After the transfusion the patient was haemodynamically stable without any further evidence of bleeding. Rest of the critical phase was uneventful. However, the platelet count did not rise as expected immediately after the critical phase. It barely passed 50 × 10^3^/μl on day 11 (6 days after the end of critical phase) at which point warfarin was restarted at a dose of 5 mg daily (Table 1). This was not accompanied with heparin due to the risk of bleeding and heparin induced thrombocytopaenia. The patient did not develop a valve thrombosis despite being off anticoagulation for 10 days (assuming the period of retained warfarin activity due to long half life since stopping warfarin is equal to the time to regain full anticoagulatory effect after resumption). The target INR was reached within 3 days of restarting warfarin and the patient was discharged on day 15. She was free of symptoms with a platelet count of 260,000/μl when reviewed in clinic one week later. Further follow up for her cardiac condition was arranged at the Institute of Cardiology, National Hospital of Sri Lanka.

**Table 1:** A summary of investigation results of the patient during the illness

**Date**	**9/7/2011**	**10/7/11**	**11/7/11**	**12/7/11**	**13/7/11**	**14/7/11**	**15/7/11**	**20/7/11**	**22/7/11**
Platelet count (X10^3^/ɥl)	95	79	44	16	07	05*	20	52	70
Packed cell volume (%)	35.5	32.7	34.3	34.8	35.1	34.1	34	33.2	34.3
Haemoglobin	10.9	11.2	12.1	11.3	11.1	10.5	10.5	8.8	9.2
White Cell Count (X10^3^/ɥl)	2.6	2.6	2.2	4.9					
AST (IU/l)			112			93			
ALT (IU/l			95			74			
PT/INR	2.2				1.1	0.96	0.97		
ESR	8								
CRP (mg/l)	12								
Blood culture	sterile								

*Platelet (4 units) and Red cell concentrate (1 unit) were transfused on 14/7/2011.

The ‘leakage’ or critical phase was from 12/7/2012 to 14/7/2012.

## Discussion

The incidence of dengue is rising in many countries and it remains a life threatening illness in the tropics. During dengue epidemics, large numbers of patients (approximately thousands) in all age groups are affected. The natural course of illness and the management approach can be complicated by underlying co-morbidities of patients.

As mentioned previously, this patient’s management during the critical or ‘leakage’ phase of the illness was complicated by two conflicting life threatening conditions; a) risk of massive internal haemorrhage or an intracranial bleed due to dengue induced thrombocytopaenia and b) the need to continue warfarin therapy to avoid a valve thrombosis of the prosthetic mitral valve. Given the lack of emergency cardiothoracic surgical facilities, a valve thrombosis could have been fatal in this patient. Therefore, withholding warfarin was a precarious balancing act to allow just enough time for the thrombocytopaenia to recover and not too long for the valve to thrombose. Decision making was further complicated by the long half life of warfarin. In the absence of guidelines or even published case reports in this regard, it was arbitrarily decided that the risk of bleeding would be significant when the platelet count dropped below 50,000/μl (a cut off value when procedures such as lumbar puncture and organ biopsies are contraindicated in thrombocytopaenic patients). Warfarin had to be stopped well before this mark. It is difficult to predict the rate of drop in platelet count in dengue as in some patients it drops drastically during the critical phase. Adding to the confusion, the critical phase is not synonymous with the period of rapid platelet drop. In other words, the platelet count may continue to fall or fail to rise even after the critical phase is over. Critical phase is only a surrogate marker for the period of rapid platelet drop. The critical / leakage phase is more correctly recognized by presence of a pleural effusion or ascites (clinically or radiologically) [2]. It is assumed that when the platelet count drops below 100,000/μl, the patient will go in to the leakage (critical) phase in the next 24 hours. Taking all in to account, we stopped warfarin when the platelet count approached 100,000/μl and anticipated an interval of at least 48 hours before it dropped below 50,000/μl. The predictions were reasonably accurate and gave us the expected time window for the action of warfarin to wear off.

Prophylactic platelet transfusions are not recommended in dengue as per national guidelines but as the patient developed evidence of active bleeding, we proceeded with transfusions to maintain the platelet count above 20,000/μl. Unexpectedly, the recovery of platelet count did not follow once the patient came out of the critical/leakage phase. It persistently hovered around 20,000-30,000/μl exceeding the 50,000/μl mark only at day 11. Warfarin was withheld till then. We preferred the platelet count to be above 100,000/μl to restart warfarin but already 10 days had lapsed without anticoagulation.

There are no case reports in published literature to our knowledge describing the experience in managing a patient with severe dengue who was also on anticoagulation after a prosthetic cardiac valve insertion (search in Pubmed with keywords ‘dengue’ and ‘anticoagulation’ or ‘warfarin’ in any field). The only case report describing a similar dilemma is by Dan et al. who describe a patient developing portal vein thrombosis following a laparoscopic cholecystectomy whose anticoagulation had to be delayed due to a concurrent dengue infection [3]. In that patient, the platelet count reached a nadir of 16,000/μl and anticoagulation was withheld for 6 days since the diagnosis of the portal vein thrombosis until the platelet count reached 125,000/μl. At that point, the patient was anticoagulated with low molecular weight heparin and later converted to warfarin.

The critical question raised by this experience is exactly how long can a person on mandatory anticoagulation for a prosthetic heart valve can be off warfarin without having a valve thrombosis. Clearly we cannot answer it in relation to dengue patients due to lack of data but indirect evidence can be drawn by experiences with patients having intracranial haemorrhages who were on warfarin [4,5]. Even then, there are only expert recommendations (no trial evidence) that suggest restarting anticoagulation within 3–10 days in patients whose anticoagulation is mandatory [6].

## Conclusion

Dengue is a life threatening illness affecting thousands of patients during epidemics. Some of them may have added complications such as being on mandatory anticoagulation. Guidelines on managing such patients are nonexistent in published literature. Our experience with this patient stresses that careful monitoring and planning of management ahead of projected changes in platelet counts can be life saving. However, the same protocol may not be valid for a second patient. Therefore it is important that clinicians share their experiences in managing such difficult patients.

## Consent and ethical background

Written informed consent was obtained from the patient for publication of this case report. A copy of the written consent is available for review by the Editor-in-Chief of this journal.

## Competing interests

The authors declare that they have no competing interests.

## Authors’ contributions

CG, CR and SS researched the background literature on the case and wrote the first draft. PK who is an infectious diseases specialist provided expert opinion on management of the patient. DW and MJ helped in clinical care of the patient. SJ contributed towards the discussions and analysis of the case. All authors reviewed the final manuscript. All authors read and approved the final manuscript.

## Authors’ information

CG (MBBS) and CR (MBBS) are registrars, in general medicine, and SS is a final year medical student, in the University Medical Unit, National Hospital, Colombo, Sri Lanka. DW and MJ (MBBS) are house officers of the unit. PK (MBBS, MD, MRCP) is Senior Lecturer and Consultant Physician of the same unit. SJ (MD, FRCP) is Professor in Medicine, the Department of Clinical Medicine, Faculty of Medicine, University of Colombo, Sri Lanka.

## Pre-publication history

The pre-publication history for this paper can be accessed here:

http://www.biomedcentral.com/1471-2334/12/272/prepub
